# Mental Influence in Disease*Read before the Rochester Pathological Society, September 16th.

**Published:** 1886-12

**Authors:** W. L. Conklin

**Affiliations:** Rochester, N. Y.


### *MENTAL INFLUENCE IN DISEASE.

By DR. W. L. CONKLIN, Rochester, N. Y.

The student of medicine very properly begins his work by seeking to obtain a thorough knowledge of the human body. In the dissecting room he observes the relative position of muscle, artery and nerve, and carefully notes the anatomical landmarks which are to aid him in future operations. In the histological and pathological laboratories he studies healthy and diseased tissues, and learns to distinguish the one from the other. He also acquaints himself with the phenomena presented by the living body, becomes familiar with the functions of its various organs and with the diseases produced by the failure or perversion of those functions. In short, his aim is to learn all he can about man—as an animal; and all will agree that the more he learns the better will he be fitted for the practice of his profession. But, in order to be fully equipped

*Read before the Rochester Pathological Society, September 16th.

for his work, he must go farther than this, and study man as man, not merely as an animal; and such study must become his life habit, if he is to attain more than the minimum of success. As well might the balance-wheel be left out of account by the machinist who would acquire a thorough knowledge of an intricate piece of machinery, as that the mind be disregarded or little thought of by the physician. The one secures steadiness and harmony of action in the various parts of a machine—wonderful, it is true, in its construction and in the work which it performs, but, after all, only a machine. The other presides over and guides to effective action a mechanism far more intricate in construction, and, when so guided, capable of producing infinitely greater and more lasting results. This two-fold nature of man ought to be kept constantly in view, for whatever our personal convictions may be in regard to the identity or non-identity of mind and matter, we are forced to deal with man as we find him—a complex being; and any failure on our part to recognize this fact, or the other fact of the close relationship existing between mind and body, will result in discomfort and disappointment at our lack of success. One of our American humorous philosophers disposed of a knotty question by cogitating thus: "What is mind? No matter. What is matter? Never mind." While I should hardly be willing to regard such conclusions as final, it is far from my purpose to attempt the discussion of so abstruse a subject as the relationship of mind and body, from a philosophical standpoint.

I wish simply to call attention to the matter as having a practical bearing on our work as physicians, hoping that this may lead to profitable discussion of what I believe to be an important subject. In the performance of every act which characterizes man as something more than an animal, the mind is the moving, controlling power. By it various lines of action are planned and carefully considered, and when a particular course has been fixed upon, the will issues its mandate, and the body, in every atom of which it is composed, obeys the command. So close is the relationship between mind and body that only when the condition of each admits of harmonious action is the man capable of effecting the highest results.

Even the machine-like involuntary acts of the body are often greatly modified by mental influence. Cases are not uncommon in which the mental depression, resulting from bad news, is sufficient completely to arrest the process of digestion; and all have noticed the effect of sudden joy or fear upon respiration and the heart's action. The following account of the method sometimes used in India for the detection of a thief is of interest, as showing the effect of fear and anxiety in checking the secretion of saliva. When a robbery has been committed, a conjurer is sent for, and if, in a few days, the property is not restored, he proceeds with his mysterious operations, one of which is as follows: The suspected are all required to masticate a quantity of boiled rice for some time, and then to spit it upon separate leaves for inspection. The conjurer examines it very knowingly, and immediately points out the culprit; the rice which he masticated being perfectly dry, while that which was masticated by the others is moistened with saliva. These are but illustrations of the many modifications of organic action which are the result of the mental influence. Alienists believe that insanity is dependent upon physical disease or abnormality. They see abundant and conclusive evidence of the close relationship between mind and body, and never think of treating the one without due attention to the other. But is the converse the case? Does the physician in general practice keep this fact as constantly in mind, and pay sufficient attention to the mental condition of his patients while treating their bodily ailments?

It has been said that the busy and successful practitioner has neither time nor inclination to pay much attention to quacks and their methods, and this is true. But perhaps he may learn at least one lesson from them. Many of them, though profoundly ignorant of the science of medicine, and as unscrupulous as ignorant, are, nevertheless, shrewd and observing; and recognizing the importance of this underlying principle of the power of mind over body, they take advantage of it in every possible way, and I believe that to this fact is due the success with which they sometimes meet in spite of their ignorance and utter disregard of the principles which lie at the foundation of all rational and lawful practice. The methods used by mind-curists, so-called faith-curists, magnetic healers, and all the rest of that class, are worthy only of condemnation; but there are other applications of the principle upon which their success, so far as they are successful, is dependent, which may well be borne in mind by the regular practitioner, and made use of by him in his efforts to combat and control disease.

I now wish to call your attention to a few well authenticated cases, which will, I hope, serve the double purpose of illustrating the influence exerted by the mind in disease, and suggesting, as well, some practical applications of the subject. It may be objected that such cases as these are exceptional, and that little, therefore, can be learned from them which will be capable of application in the daily routine of practice. But I believe that mental influence which is the same in kind, if not in degree, is present in many of the cases with which we meet, and that it should be taken into account as an important factor in the problem of how best to treat such cases.

Hope is a medicine, the use of which is clearly indicated in a great variety of diseases; and so little does it possess of troublesome incompatibility or unchangeableness of form, that, as has been said, "It can concentrate its healing virtue in a homeopathic globule or diffuse it through all the multitudinous baths, douches and wet bandages of a hydropathic establishment." The disastrous effects of a loss of hope, as well as its wonderful efficacy as a remedial agent, are illustrated by the following incident: During the seige of Breda, in 1625, many of the soldiers in the army of the Prince of Orange were prostrated with scurvy. Patients had lost all hope, and this, says Dr. Frederick van der Mye, who was present, was the most terrible circumstance of all, and the evident cause of the great mortality which attended the disease. At length it was announced that an infallible remedy had been discovered, and that it would be given to all who were sick. Hope came back to those who had been hopeless; and the administration, in minute doses, of a few vials of sham medicine was followed by astonishing results. Such as had not moved their limbs for months, says the account, were seen walking in the streets, sound, straight and well. Surely if hope, growing out of the effect on the imagination of the administration of a placebo, is capable of producing such results, the physician will do well to avail himself, so far as possible, of its aid, while making intelligent use of remedies which he believes to be in themselves effective; and is it not best to retain this powerful ally, as long as possible, by concealing the fact from patients whom we believe to be dangerously ill, so long as we have the least hope of their recovery? "Hope deferred maketh the heart sick," and the loss of hope is not unfrequently the cause of fatal sickness. It is said that criminals who have received a life-sentence often rally from severe attacks of illness with unusual difficulty, and that they are much more liable to a relapse from the slightest cause, than are those who are sustained and strengthened by the cordial of hope. The direful results of hopelessness are strikingly illustrated by a disease with which we are, for the most part, at least, happily unfamiliar. I quote from a description of the disease, written by an English physician, forty years ago. "It begins by indulgence in despondency, then follows loss of appetite, constant pain in the stomach, difficulty in breathing, paleness of the face and palms of the hands, whiteness of the tongue, with inky spots on it, white lips, and inability to move. Then the white of the eyes becomes glassy, the skin turns of an olive color and cold to the touch, water collects in every part of the body and the sufferer cannot breathe, except in an erect position. The glands then become inflamed, the liver hardened, and the blood, poor, vapid and colorless, no longer stimulates the heart, and death soon terminates the scene. This malady is dignified by the title Cachexia Africana, because, alas! it has killed thousands on thousands of the children of Africa, when forced from 'home and all its pleasures.'" The physician can often do much to inspire his patients with hope and courage. Having made a diagnosis he knows with what he has to deal, and knows, also, that in a large majority of cases of acute disease the prognosis is good. He believes, as well, that in many chronic cases a judicious use of remedies will be followed by an improvement, at least, in the condition of his patient. The patient, on the contrary, knows little about his real condition, and perhaps is torturing himself with the fear that his sickness will terminate fatally, when, in reality, there is little danger of such a result. What wonder, then, that a few cheering reassuring words do the sick man a world of good; and is it not one of the pleasant duties of a physician to make use of such a potent remedy, when he can do so in honesty.

The course and issue of disease is often influenced to so great a degree by the mental make-up of the patient that the latter should be taken carefully into account in the prognosis. A strong will power in the patient affords valuable aid to the physician, while the lack of it often does much to render his best efforts of no avail. It is related of Andrew Crosse, the electrician, that he was once severely bitten by a cat, which, on the same day, died of hydrophobia. He succeeded in dismissing all fears from his mind, and no sign of the dread disease appeared until the end of three months, when he suddenly felt severe pain in his arm, accompanied by intense thirst. A strong spasm shot across his throat, and then the terrible conviction came to his mind that he was about to fall a victim to hydrophobia. He says that for an hour he suffered almost every conceivable agony of mind and body, and then, believing it to be his only hope, he determined to make a desperate effort to overcome the disease. Taking his gun, he started out in search of game. None was found, but all day long he kept going, and, though suffering severely, his determination to get the better, if possible, of the fearful malady, did not diminish in strength. On reaching home at night, he felt much better, and, in three days, the pain and other symptoms had entirely disappeared. In striking contrast with this case is one recorded by Dr. John Moore. A woman had her dress bitten by a dog. She had heard of hydrophobia, and immediately fancied that she had it; and, what is more surprising, she died while manifesting symptoms so like those of canine madness that skillful physicians could not discover any difference.

Dr. J. M. Buckley, says that "as long ago as the time of John Hunter, it was established by a variety of experiments, and by his own experience, that the concentration of attention upon any part of the human system affected first the sensations, then produced a change in the circulation, next a modification of the nutrition, and finally a change in structure;" and there can, I think, be little doubt that such organic change sometimes results from mental influence. A writer in *Littell's Living Age* records a case in point, the facts of which are, in substance, as follows: A young lady, of rather nervous temperament, imagined that a bristle from her tooth brush had lodged in her throat. Careful examinations were made by the family physician and others, but all failed to find the bristle, and all assured the young lady that she must be mistaken in regard to the matter. She refused to believe them, however, and after a time there was actual inflammation present, as if from the irritation of a foreign body. Matters went from bad to worse until, at last, a young surgeon was called, who, after hearing the history of the case, adopted a new plan of treatment. After a thorough examination he told his patient that she was quite right and that he had at last discovered the offending bristle, but it would be necessary for him to go to his office for the best instrument with which to remove it. At the office he carefully concealed a bristle within the instrument, and on returning introduced both with great care, into his patient's throat. With enough of a pinch of the sensitive mucous membrane to give her an idea that something was being done, the bristle was withdrawn and exhibited in triumph to the young lady, who now had the intense satisfaction of supposing that she had been right and all her former physicians wrong. The inflammation quickly subsided after the removal of its mental cause, and the young surgeon's wonderful skill was heralded far and near.

In cases termed "hysterical" we see evidence, perhaps more than in any others, of the close relationship between mental and bodily conditions, and the former as well as the latter should be duly considered in the treatment. Often an abnormal mental condition, together with the disturbance of bodily function which it has produced, will quickly disappear under the influence of some powerful mental impression. This truth is well illustrated by the following incident, though such heroic treatment would hardly be suited to private practice: "The great Boerhaave had a number of patients seized with epileptic fits, in a hospital, from sympathy with a person who fell down in a convulsion before them. This physician was puzzled how to act, for the sympathetic fits were as violent and obstinate as those arising from bodily disease. But, reflecting that they were produced by an impression on the mind, he resolved to eradicate them by a still stronger impression, and so directed hot irons to be prepared and applied to the first person who subsequently had a fit. The consequence was not a person was seized afterward."

As careful and accurate an observer as J. Milner Fothergill has noticed particular mental characteristics present in various diseases. He says much mental instability is found among sufferers from chronic heart disease. A very old patient who was the subject of aortic obstruction became remarkably polite when the results of the cardiac lesion were very marked, a mental attitude far removed from that assumed at other times. He has observed, however, that usually a totally opposite character of change is produced, and the effect is to cause the mental operations to be imperfect, unsustained and unequal; while there are present suspicion, doubtfulness, vacillation and caprice. The gouty patient is a compound of irritability and suspicion, bad temper and anxiety. The victim of cancer shows a sullen and defiant submission to the inevitable. There is rarely any active and positive attempt made by the sufferers themselves to avert their doom. The mental attitude of pyæmia is that of absolute indifference. In diabetes millitus there is a condition of mental languor and depression which is as marked as the muscular lethargy and lassitude manifested by sufferers from that disease. And it is no doubt true that not unfrequently a more or less morbid mental condition is one of the results of severe or long-continued sickness. This condition reacting upon the body, tends to interfere with a return to physical strength and soundness. In this way a vicious circle is formed which must be broken in upon before health can be restored, or even a marked improvement effected in the condition of the patient. Such patients are often advised to try a change of air, as the phrase goes, and this is sometimes followed by decided improvement. But is not the improvement due, in a large degree at least, to the mental rest and recuperation resulting from a change in the general trend of thought and feeling? While even a brief sojourn in a distant state is out of the question with the majority of our patients, many of them would not look upon a visit of a few days or weeks in duration to an adjoining town or county as an impossibility, and I believe that often as much improvement would result from the latter change as from the former. The physician may sometimes render an invaluable service by insisting on the importance of an entire change of surroundings for patients, who, worn out by disease or undue mental strain, are in danger of lapsing into a condition of chronic invalidism or the still sadder condition of mental derangement.

Mind and body, then, bear a close relationship to each other, and disease of the latter is sometimes caused, very often greatly modified, by the condition of the former. If this fact be kept in mind, and its importance duly considered in deciding on a plan of treatment, I believe that success will often follow where failure would have resulted had no account been made of the modifying effects of mental influence.

